# Living la Vida T-LoCoH: site fidelity of Florida ranched and wild white-tailed deer (*Odocoileus virginianus*) during the epizootic hemorrhagic disease virus (EHDV) transmission period

**DOI:** 10.1186/s40462-020-00200-2

**Published:** 2020-03-16

**Authors:** Emily T. N. Dinh, Allison Cauvin, Jeremy P. Orange, Rebecca M. Shuman, Samantha M. Wisely, Jason K. Blackburn

**Affiliations:** 1 Spatial Epidemiology & Ecology Research Laboratory, Department of Geography, University of Florida, Gainesville, FL USA; 2 Emerging Pathogens Institute, University of Florida, Gainesville, FL USA; 3 Department of Wildlife Ecology and Conservation, University of Florida, Gainesville, FL USA; 4 Florida Fish and Wildlife Conservation Commission, Gainesville, FL USA

**Keywords:** White-tailed deer, Farmed deer, Ranched deer, Home range, Site fidelity metrics, T-LoCoH, Disease risk

## Abstract

**Background:**

Epizootic hemorrhagic disease virus (EHDV) is a pathogen vectored by *Culicoides* midges that causes significant economic loss in the cervid farming industry and affects wild deer as well. Despite this, its ecology is poorly understood. Studying movement and space use by ruminant hosts during the transmission season may elucidate EHDV ecology by identifying behaviors that can increase exposure risk. Here we compared home ranges (HRs) and site fidelity metrics within HRs using the T-LoCoH R package and GPS data from collared deer.

**Methods:**

Here, we tested whether white-tailed deer (*Odocoileus virginianus*) roaming within a high-fenced, private deer farm (ranched) and native deer from nearby state-managed properties (wild) exhibited differences in home range (HR) size and usage during the 2016 and 2017 EHDV seasons. We captured male and female individuals in both years and derived seasonal HRs for both sexes and both groups for each year. HRs were calculated using a time-scale distance approach in T-LoCoH. We then derived revisitation and duration of visit metrics and compared between years, sexes, and ranched and wild deer.

**Results:**

We found that ranched deer of both sexes tended to have smaller activity spaces (95% HR) and revisited sites within their HR more often but stayed for shorter periods than wild deer. However, core area (25% HR) sizes did not significantly differ between these groups.

**Conclusions:**

The contrast in our findings between wild and ranched deer suggest that home range usage, rather than size, in addition to differences in population density, likely drive differences in disease exposure during the transmission period.

## Background

The Cervidae (deer) farming industry is one of the fastest growing industries in the rural United States, generating an estimated $8.0 billion in economic activity annually; the majority of farms produce or manage white-tailed deer (WTD; *Odocoileus virginianus*) [1]. Infectious diseases affecting these deer present a major industry challenge. One important infectious pathogen is epizootic hemorrhagic disease virus (EHDV), an orbivirus within the family Reoviridae vectored by female *Culicoides* biting midges (Diptera: Ceratopogonidae). Infection with EHDV can lead to epizootic hemorrhagic disease (EHD), which deer often survive, but the disease can cause disability for extended periods of time because of lameness and emaciation caused by loss of appetite [2, 3]. In addition, infected animals are vulnerable to secondary respiratory infections and can develop extensive organ damage [2, 4, 5]. Severe hemorrhage-induced death can occur within 1–3 days of infection [2, 6, 7]. EHDV has been detected in a variety of wild and domestic ruminant species in almost every state in the U.S. [8], with high incidence of infection but low mortality in the southeast [6]. Although EHDV is widespread throughout the U.S., treatment is currently lacking, and control strategies are limited. The vaccines that are presently commercially available do not induce a significant, measurable, robust antibody response in WTD inhabiting a high-fenced outdoor enclosure [9, 10] and their delivery to deer roaming large properties is untenable, as these vaccines are injectable [11]. *C. sonorensis*, the only confirmed vector of EHDV in the U.S., is rare in southeast and its life history is poorly known; knowledge on the biology and ecology of the *Culicoides* genus is overall lacking [12]. Prevention will always remain a necessary measure against EHD but is limited by the lack of knowledge of virus-vector-host interactions [13]. Consequently, EHD is a major concern for deer farmers.

WTD roaming on high-fenced deer farms in northern Florida have been found to have higher exposure to EHDV than wild deer inhabiting state-managed properties nearby [14]. However, little is known about what may drive those differences in exposure. Density can be a major driver of disease prevalence in a population, as exemplified in a Reunion Island study where sheep, goat, and cattle herds from regions with relatively high cattle density had high EHDV seroprevalence [15]. Cauvin et al. [14] examined the higher density of a farmed deer population vs. wild could explain greater EHDV exposure amongst farmed deer. However, other factors besides population density are important for disease transmission. Quantifying how a host animal uses space during the EHDV transmission season can be important for relating animal movement behaviors to disease risk and may elucidate disease ecology by revealing when and where transmission may occur [16, 17]. Several published studies linking animal movement to disease risk exist for indirect transmission of environmentally mediated pathogens, where animals contact the pathogen during foraging or exploring. For example, Brook et al. [18] quantified co-mingling of elk and WTD with cattle to assess the potential for bovine tuberculosis transmission and found that it was higher from WTD, as no GPS-collared elk locations overlapped with cattle winter feeding areas but about one-fifth of GPS-collared WTD locations did. Fewer studies have evaluated movement ecology relative to vector-borne diseases; many of those focus on bird-mosquito interaction. In a study on West Nile Virus (WNV), crow movements were monitored to estimate how birds may move the virus between locations/habitats (foraging and roosting areas) [19]. That same study characterized bird behaviors during active viremia and noted that lethargic activity with limited insect avoidance could lead to increased mosquito feeding events and subsequent transmission. In another study, Janousek et al. [20] employed transmitters to link the roosting behaviors of seven North American avian WNV host species with host-seeking mosquito abundance. They found that mosquito abundance increased with roosting height and there were fewer mosquitoes per bird at communal roosts. Therefore, variation in fine-scale habitat use by hosts may influence vector-host interactions.

Since it is unknown if wild and ranched deer exhibit similar movement and site fidelity behaviors, we employed a similar framework here to begin to reveal differences between ranched and wild deer that may lead to differential disease risk. Understanding behaviors may identify intervention opportunities to disrupt transmission, such as modifying deer habitat or feeding locations that reduce host-*Culicoides* interaction. As a starting point, we compare the seasonal HR and site fidelity behaviors of free-ranging ranched and wild WTD during the EHDV transmission season. In a serological study of 27 individual free-ranging ranched deer and 53 wild deer in the same study areas, we found that the ranched population had significantly higher EHDV seroprevalence and antibody titers than their wild counterparts [14]. Movement behaviors may increase the magnitude of the infection risk, so in this study, we examine differences in behaviors between ranched and wild Florida WTD.

Since home ranging behaviors can influence when and how often an animal meets an infectious vector, the objectives of our study were to determine whether 1) free-ranging WTD in a privately owned and managed high-fenced preserve (ranched) and native WTD from nearby state-managed properties (wild) exhibited differences in HR size (define here by 95% activity space and 25% core area) and 2) usage (i.e., behaviors within the HR) during the 2016 and 2017 EHDV transmission seasons. We hypothesized that wild deer of both sexes would have larger HRs but fewer site revisitations and longer duration of stay at sites, as wild deer are not confined by property boundaries, exist at lower densities, and do not have point source feeders that may concentrate behavior. Larger HRs but greater site fidelity by wild WTD may allow them fewer interactions with infectious vectors, thus possibly explaining lower EHD burden amongst wild deer populations.

## Methods

### Study areas

**Fig. 1:**
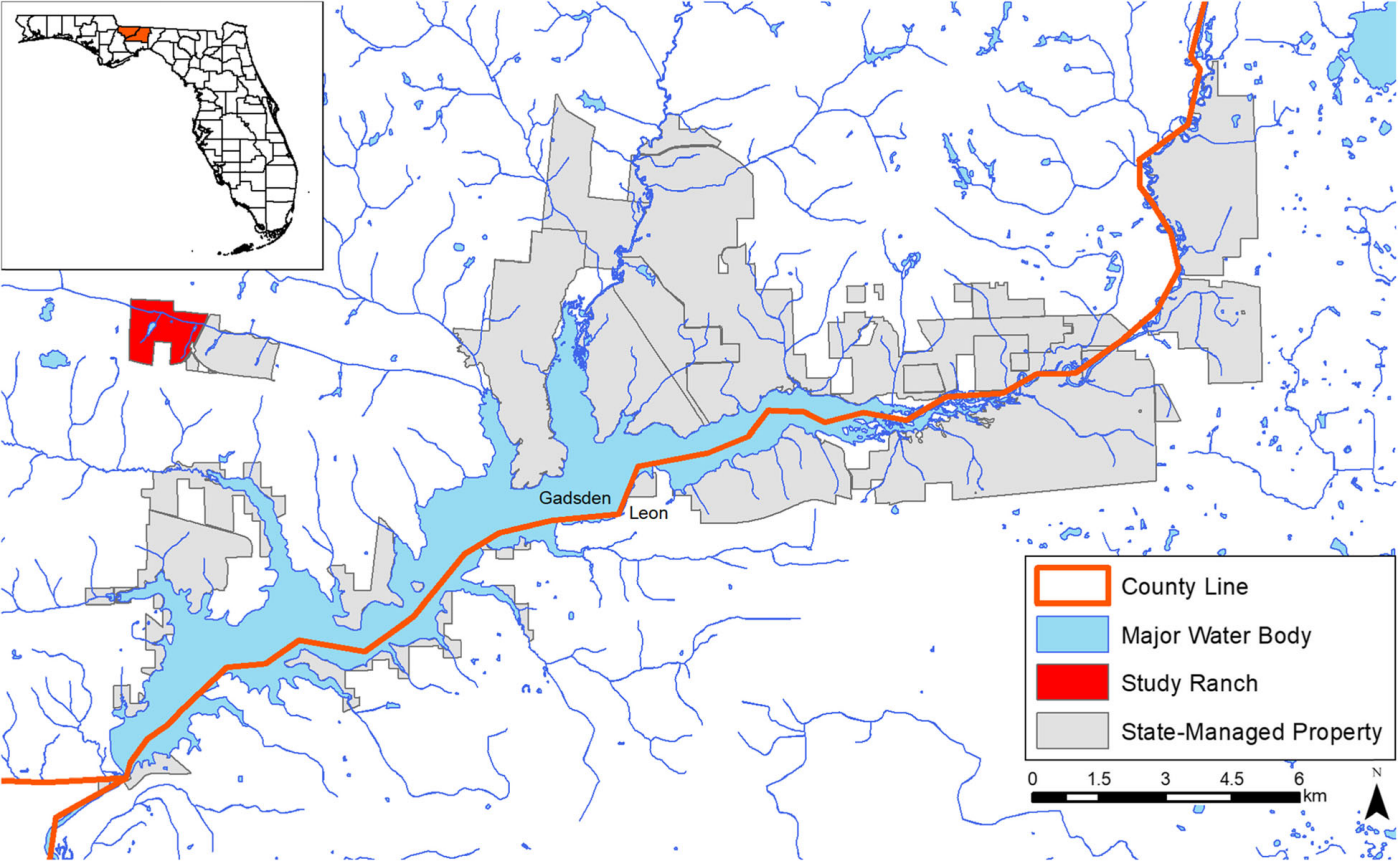
Location of the study deer farm and nearby state-managed properties where wild deer were studied in Gadsden & Leon counties, Florida

**Fig. 2:**
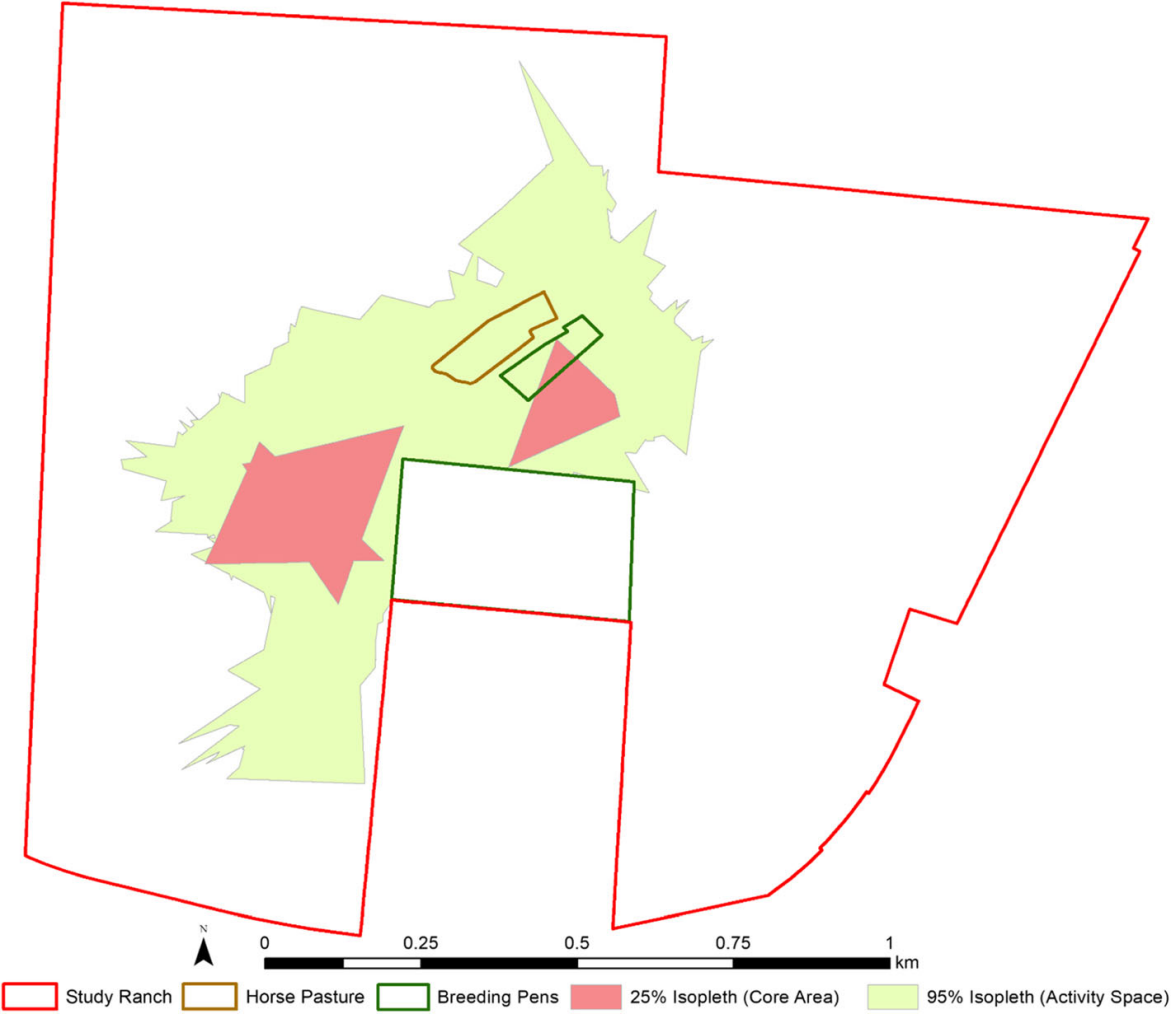
Isopleths generated for one ranched deer (OV063) with the T-LoCoH package in R

Our study on ranched deer was conducted within an approximately 200-ha privately owned, high-fenced preserve in Gadsden County, Florida (Fig. 1). The property area included adjacent deer breeding pens (Fig. 2). The preserve contained ≃130–150 unpenned WTD and ≃150 non-native bovids and cervids that roamed the property. Bovids inhabiting the study ranch included 30–40 blackbuck antelope (*Antilope cervicapra*), 6–8 nilgai (*Boselaphus tragocamelus*), 6–8 scimitar-horned oryx (*Oryx dammah*), and 7–9 gemsbok (*Oryx gazella*). Cervids besides WTD included 40 axis deer (*Axis axis*), 19–22 elk (*Cervus canadensis*), sika deer (*Cervus nippon*), and sika-elk hybrids, 12–24 fallow deer (*Dama dama*), and 7–9 Père David’s deer (*Elaphurus davidianus*) [21]. Many of these non-native animals can become infected with EHDV [5], but it is unknown what role they play in maintaining the natural cycle of EHDV. The array of animals on the private ranch yielded approximately 1.48 animals/ha, with an estimated 0.78 WTD/ha [14]. The dominant landscape on the property was hardwood hammock. Upland short leaf pine species such as lobolly (*Pinus taeda*) were also a prominent feature on the farm. The property was managed with food plots and 12 stationary (point source) supplemental protein feeders regularly filled by ranch staff.

Wild deer were studied on state-managed properties (total area approximately 7800 ha) within Gadsden and Leon counties, near the study farm (Fig. 1). These properties were managed by the Florida Fish and Wildlife Conservation Commission (FWC) and the Florida Forest Service. Their management objectives were multiple use: human recreation, timber harvest, and environmental needs. The landscape on these properties consisted of a mixture of hardwood hammock, mesic flatwoods, upland pine (*Pinus elliottii* and *P. palustris*), and sandhill. The pine on the state-managed properties was either naturally regenerated or in even-aged stands. The FWC estimated the density of wild WTD in our study areas to be ≃0.08 animals/ha [14].

### Telemetry data collection and preparation

On the study ranch, we captured 8 male and 7 female WTD (*n* = 15) in 2016 and 3 male and 3 female WTD (*n* = 6) in 2017 using chemical immobilization delivered via dart gun from April–June each year. We fitted ranched deer with GPS collars (model 3300 L or 3300S, Lotek, Newmarket, ON, Canada; or model G2110E2 (NeoLink), ATS, Isanti, MN, U.S.A.). Similarly, on state-owned properties, we captured 3 male and 5 female WTD (*n* = 8) in 2016 (May–July) and 7 males and 11 female WTD (*n* = 18) in 2017 (January–March) and fitted them with ATS collars (G2110E (NeoLink), ATS, Isanti, MN, U.S.A.). Captures were typically conducted during crepuscular hours. We programmed collars to record GPS fixes every 30 to 60 min until September–November each year. Of all deer captured, four wild and one ranched deer were collared in both study years to acquire enough GPS fixes for statistical power.

Prior to analysis, we removed individuals that had fewer than the mean number of days tracked during the EHDV transmission season minus the standard deviation for that year: 127–38 d and 158–39 d for 2016 and 2017, respectively. Then, we thinned bursts, several GPS points recorded within a few minutes often caused by communication errors between a GPS collar and satellite, to avoid extraneous clustering of movement data [22]. We next resampled animals that had 30-min GPS intervals to a common interval of 60 min to enable comparison between individuals. Here, we define summer to fall (May–October) as the EHDV transmission season in the southeastern U.S. [8]. Thus, for this study, we subsampled all GPS data from 11:00 PM EST on 30 April to 11:00 PM EST on 30 October. Capture information, tracking periods, and individuals excluded from analysis are listed in Table S1.

### Home range size & site Fidelity estimation

In this study, we focused on Johnson’s second and third orders of selection, which correspond to an individual animal’s home range (HR) and the individual’s usage of various habitat components within its HR, respectively [23]. The HR can be considered an account of the relative frequency distribution of locations used by an animal [24], where the 95% subset, or activity space, is a summary of this distribution and the 25% subset, or core area, is the proportion of data such that the probability of use of an area is disproportionately high [25]. Understanding the second and third orders of selection behavior exhibited by WTD in the epidemiology and ecology of EHDV can enable deer managers to discover temporally and geographically predictable movement patterns that may expose deer to infectious vectors and thus learn where and when to target subsequent surveillance and intervention efforts [26].

To measure second then third order of selection behavior displayed by WTD in our study, we first estimate the seasonal home range, then calculate site fidelity metrics that relate long periods of time in or frequent usage of areas within the HR. We used the ‘T-LoCoH’ package (version 1.40.05) in R to define the home range for each deer [22, 27]. T-LoCoH is a non-parametric method for constructing HRs from a set of locations by aggregating local convex polygons constructed around each point and its nearest neighbors. T-LoCoH balances temporal autocorrelation in GPS data by incorporating a time-scaled distance (TSD) metric, which measures the distance between two points in both space (X/Y coordinates) and time (timestamps) to translate a unit of time into a unit of distance [28, 29]. The time and space components of TSD are weighted by *s*, a dimensionless scaling factor of the maximum theoretical velocity (*v*_*max*_), or distance, an individual animal could have traveled during the time interval [28]. Here we selected an *s* value such that 60% of the polygons were time-selected for each deer [22, 29]. We used the *a*-method for identifying nearest neighbors, computing with the auto.a() function an *a* value for each deer to allow for variation between individuals. We chose the 25 and 95% aggregations of polygons, or isopleths, to represent the core area and activity space, respectively [25] (Fig. 2). To quantify site fidelity within HRs, or third-order selection behavior [23], we calculated revisitation (NSV; number of separate visits) and duration (MNLV; mean number of locations per visit) metrics for each polygon based on an inter-visit gap (IVG; the time that must pass for separate visits) of 12 h. Resulting polygons and isopleths were exported as shapefiles to extract the NSV and MNLV measurements from each polygon and remove breeding pens that were inaccessible to unpenned animals roaming the property from all isopleths before recalculating their sizes in ArcGIS version 10.3.1 [30]. We did not remove the single ≃0.8 ha pen or horse pasture from the isopleths because ranched deer were often observed around the perimeter of the previous and could access the latter. The isopleth sizes and NSV and MNLV metrics for each polygon were exported as CSVs for statistical analysis in R version 3.4.0 [31].

We used the Wilcoxon rank sum test to discern significant differences (*α* = 0.05) in the sizes (ha) of the 1) 95% activity spaces and 2) 25% core areas; and a random 75% sample of each deer’s 3) NSV and 4) MNLV metrics between ranched and wild WTD grouped by sex during the EHDV season [17, 32, 33]. We applied the test to each metric in R, excluding one ranched female and two wild female deer tracked in 2016 and two wild female deer tracked in 2017 due to insufficient sampling during the EHDV transmission season. Sample sizes for differences in NSV and MNLV are detailed in Table S2. We generated boxplots with the ‘ggplot2’ package in R to illustrate the results of our statistical analysis [34].

## Results

Wilcoxon tests for differences in activity space size between ranched and wild WTD of both sexes were statistically significant in both study years (2016 female *p* = 0.0238, 2016 male *p* = 0.0489, 2017 female *p* = 0.0091, 2017 male *p* = 0.0333). The mean size of ranched female deer activity spaces was 28.73 ± 3.94 ha in 2016 and 21.37 ± 1.32 ha in 2017, while wild female deer had activity spaces that averaged 49.94 ± 10.98 ha in 2016 and 52.10 ± 3.19 ha in 2017. Ranched male deer activity space averaged 33.95 ± 3.69 ha in 2016 and 36.82 ± 7.92 ha in 2017 while wild male deer averaged 96.25 ± 29.51 ha in 2016 and 163.91 ± 38.22 ha in 2017. In general, wild WTD of both sexes had larger activity spaces than ranched WTD. However, core area size was not significantly different between these four groups in both study years (2016 female *p* = 0.1667, 2016 male *p* = 0.1939, 2017 female *p* = 0.1, 2017 male *p* = 0.1167). Ranched female deer had an average core area size of 4.04 ± 0.83 ha in 2016 and 4.39 ± 0.27 ha in 2017. Wild female deer had an average core area size of 6.03 ± 1.44 ha in 2016, 6.33 ± 0.67 ha in 2017. Ranched male deer encompassed a mean core area size of 4.26 ± 0.80 ha and 4.32 ± 1.40 ha in 2016 and 2017, respectively while wild male deer had averages of 9.72 ± 3.54 ha in 2016 and 14.50 ± 3.90 ha in 2017.

**Fig. 3:**
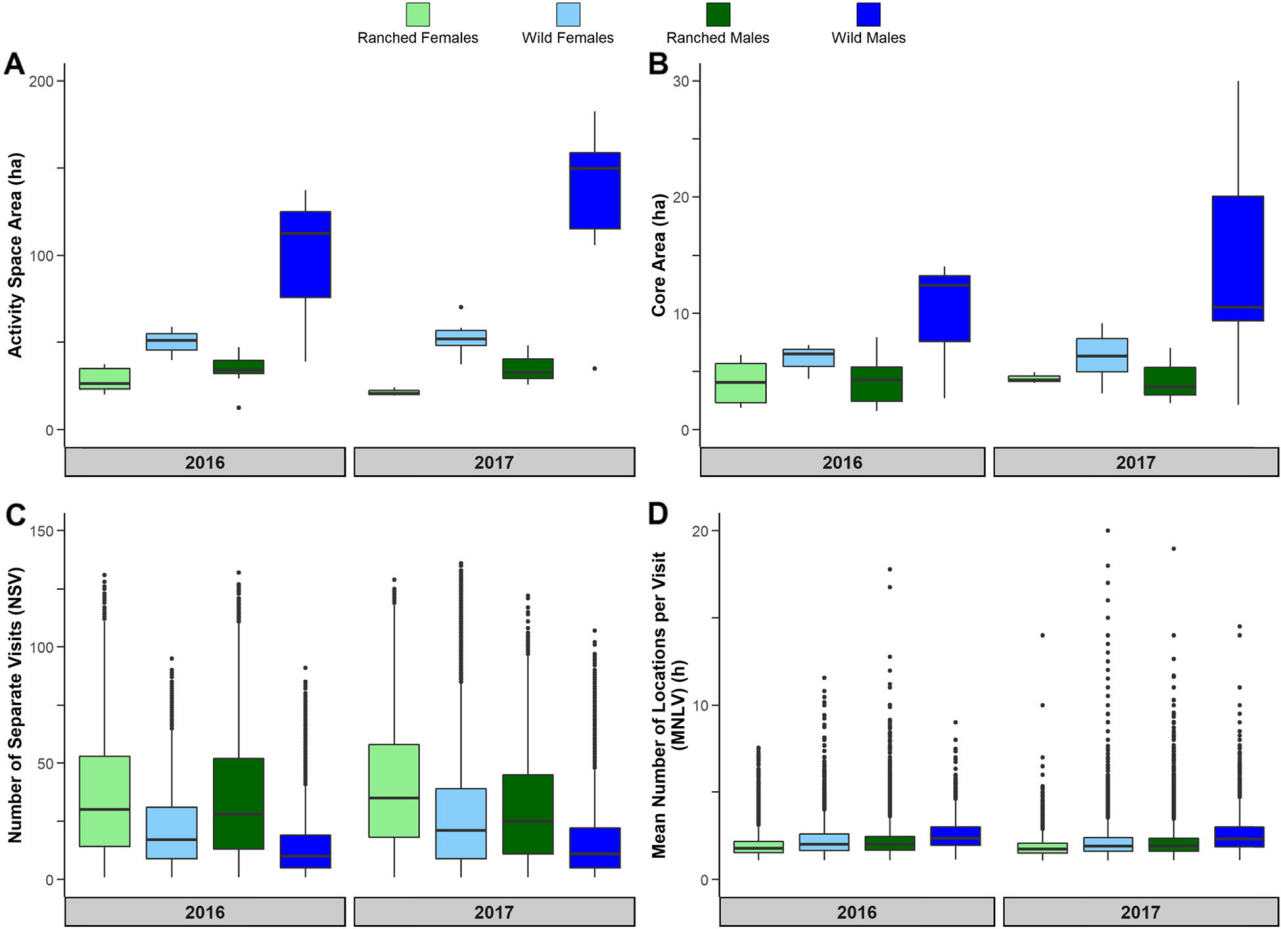
Plot of the descriptive statistics summarizing differences in home range behavior between wild and ranched deer: **a** the size of the activity space (95% isopleth), **b** the size of the core (25% isopleth), **c** revisitation of polygons, and **d** duration of stay within polygons

All tests for site fidelity metrics were statistically significant amongst the 4 groups of deer each study year (Table 1, all *p*-values < 2.2 × 10^− 16^). Ranched female deer revisited sites within their HRs an average of 35.90 ± 0.19 times in 2016 and 40.73 ± 0.27 times in 2017, while wild female deer revisited sites an average of 22.17 ± 0.17 and 27.19 ± 0.12 times in 2016 and 2017, respectively. The mean number of site revisitations for ranched male deer was 35.34 ± 0.18 times in 2016 and 29.81 ± 0.21 times in 2017, while wild male deer had a mean of 14.32 ± 0.14 and 15.63 ± 0.09 times in 2016 and 2017, respectively. Ranched female deer had a mean duration of stay at a site in their HR of 1.96 ± 0.00 h in 2016 and 1.86 ± 0.01 h in 2017 while wild female deer stayed for an average of 2.25 ± 0.01 h and 2.16 ± 0.01 h in 2016 and 2017, respectively. Ranched male deer stayed for an average of 2.19 ± 0.01 h in both study years while wild male deer stayed for 2.62 ± 0.01 h in 2016 and 2.64 ± 0.01 h in 2017, respectively. In general, ranched WTD of both sexes revisited sites within their HRs more often but stayed for shorter periods of time each visit. In contrast, wild WTD of both sexes visited sites within their HRs less often but remained at sites for longer periods of time. Results are summarized in Fig. 3 and Table 1.

**Table 1:** Wilcoxon rank sum test results for differences in home range behavior between ranched and wild deer during the 2016 & 2017 EHDV seasons. Significant differences are denoted by *

Year	Comparison	Ranched Mean ± SE	Wild Mean ± SE	Wilcoxon rank sum W	***p***-value
2016	Female 95% Activity Space (ha)	28.73 ± 3.94	49.94 ± 10.98	0	0.0238*
	Female 25% Core Area (ha)	4.04 ± 0.83	6.03 ± 1.44	3	0.1667
	Male 95% Activity Space (ha)	33.95 ± 3.69	96.25 ± 29.51	2	0.0489*
	Male 25% Core Area (ha)	4.26 ± 0.80	9.72 ± 3.54	5	0.1939
	Female Revisitation	35.90 ± 0.19	22.17 ± 0.17	73,058,000	< 2.2 × 10^− 16^*
	Female Duration (h)	1.96 ± 0.00	2.25 ± 0.01	42,899,000	< 2.2 × 10^− 16^*
	Male Revisitation	35.34 ± 0.18	14.32 ± 0.14	89,829,000	< 2.2 × 10^− 16^*
	Male Duration (h)	2.19 ± 0.01	2.62 ± 0.00	41,042,000	< 2.2 × 10^− 16^*
2017	Female 95% Activity Space (ha)	21.37 ± 1.32	52.10 ± 3.19	0	0.0091^♦^
	Female 25% Core Area (ha)	4.39 ± 0.27	6.33 ± 0.67	3	0.1
	Male 95% Activity Space (ha)	36.82 ± 7.92	163.91 ± 38.22	1	0.0333*
	Male 25% Core Area (ha)	4.32 ± 1.40	14.50 ± 3.90	3	0.1167
	Female Revisitation	40.73 ± 0.27	27.19 ± 0.12	148,790,000	< 2.2 × 10^− 16^*
	Female Duration (h)	1.86 ± 0.01	2.16 ± 0.01	91,440,000	< 2.2 × 10^− 16^*
	Male Revisitation	29.81 ± 0.21	15.63 ± 0.09	120,810,000	< 2.2 × 10^− 16^*
	Male Duration (h)	2.19 ± 0.01	2.64 ± 0.01	59,718,000	< 2.2 × 10^−16^*

## Discussion

In this study, we tested whether seasonal HR size (second order selection behavior) and site fidelity (third order selection) behaviors differed between ranched and wild WTD during the 2016 and 2017 EHDV transmission seasons. In both study years, we found that ranched deer had smaller activity spaces than wild deer, but we detected no significant differences in the size of the core areas in any group (second order selection). However, we did detect significant differences in how ranched and wild deer utilized their HRs (third order selection) during the risk period. Broadly, ranched WTD of both sexes tended to revisit individual sites within their HR more often but stay at any given site for shorter periods of time than wild deer. The ranched WTD in our study may have had smaller activity spaces compared to wild WTD due to the association of small HRs with high population densities, though there is little evidence to indicate whether this is a causal relationship. A more possible explanation is that ranched animals likely have greater/easier resource availability (perhaps due to their access to supplemental protein feed) compared to wild animals, thus reducing the ranched animals’ need to range a larger area to survive. Furthermore, our results suggest that the greater exposure to EHDV amongst ranched WTD in comparison to wild WTD observed by Cauvin et al. [14] may be due to dissimilar home range use rather than size. The site fidelity behaviors occurring within ranched deer core areas may affect how the animals interact with midges and their subsequent exposure to EHDV. In addition to population density, the movement data we collected in this study are likely additive effects that increase pathogen exposure.

Although this study is limited regarding its applicability to other deer populations that experience EHD in the US, features of the deer populations we studied here are like those in other studies. For example, supplemental feeding of wildlife is a common management practice that can increase potential for disease transmission [35]. The supplemental protein feeders on the study ranch may have adverse effects on WTD health by concentrating susceptible individuals at sites containing infectious midges. Wild deer may receive supplemental food resources from private landowners and/or FWC, but it is difficult to know to what extent (though it is less than quantities provided on high fenced farms). Moreover, higher host densities have been associated with increased brucellosis seroprevalence in wild elk (*Cervus elaphus*) [36]. Similarly, the high density of animals on the study ranch may increase local host density around the feeders, which could increase transmission potential. More frequent, shorter visits to sites by ranched deer and their higher levels of EHDV exposure compared to wild deer may be explained by intraspecific and/or interspecific competition with exotic species at supplemental protein feeders. Ranched WTD may experience greater levels of stress and reduced immunity as they intensely compete with an artificially inflated population of exotics and conspecifics for food [37]. Thus, HR use behaviors in a dense population, not just population density alone, may drive greater disease prevalence amongst ranched WTD.

Strategically locating supplemental feed sites or increasing the number of feeders may help mitigate disease risk. For example, supplemental feed sites in areas with high percentages of hardwood forests in Michigan were associated with decreased risk of bovine tuberculosis in wild WTD [38]. Deer ranchers in Florida may be able to lower the risk of their animals contracting EHDV by altering the placement of their feeders to areas less suitable for *Culicoides* vectors and/or by encouraging animals to disperse from large concentrations by increasing the number of feeders within a property. This can also apply to wild WTD that visit food plots on state-managed properties; those plots may be near or in habitats favorable to *Culicoides*, so changing the location of the plots and/or adding more may help direct deer away from vector habitat(s).

## Conclusions

In conclusion, studying the movement of host animals during a disease transmission period can reveal what behaviors may increase exposure risk and then enable managers to mitigate disease risk by modifying animal behavior. We did not observe significant differences in core area size between wild and ranched deer during the 2016 and 2017 EHDV transmission seasons. Rather, we found that these two populations of deer differed in their behaviors within their home ranges. We infer from these findings that smaller home ranges and greater site revisitation exhibited by ranched deer may drive higher exposure to EHDV; this may be exacerbated by higher host density of ranched animal, which is possible through supplemental feed [14]. Deer that revisit sites populated by infectious *Culicoides* increase the frequency of vector-host interactions and provide vectors with a greater abundance of susceptible blood meal hosts. Consequently, wildlife managers and deer farmers should consider management strategies that affect resource selection and foraging decisions of WTD. One future direction of this research is to investigate whether space use by WTD is primarily driven by resource distribution and availability or intraspecific (i.e., exploitive) and/or interspecific competition (e.g., interference with exotic species on the study ranch or with wild boar, *Sus scrofa,* on public lands) [39]. Additionally, measuring vector incidence at sites of high deer revisitation would reveal whether, when, and where animals encounter infectious vectors there and locate target areas for disease control and prevention efforts.

## Supplementary information

**Additional file 1: Table S1.** Deer collared for this study. ^†^30 Apr – 30 Oct = 183 days. ^×^Individuals excluded from home range behavior analysis. ^Individuals collared beyond 31 Oct 2017.

**Additional file 2: Table S2.** Sample sizes for testing significant differences in revisitation and duration between groups.

## Data Availability

The datasets generated and/or analysed during the current study are not publicly available due private landowner privacy agreement but are available from the corresponding author on reasonable request.

## Abbreviations

EHDV: Epizootic hemorrhagic disease virus
FWC: Florida Fish and Wildlife Conservation Commission
GPS: Global positioning system
HRs: Home ranges
IVG: Inter-visit gap
MNLV: Mean number of locations per visit
NSV: Number of separate visits
WTD: White-tailed deer

## References

[1] Anderson DP, Outlaw JL, Earle M, Richardson JW (2017). Economic impact of U.S. deer breeding and hunting operations.

[2] Southeastern Cooperative Wildlife Disease Study (2013). Hemorrhagic disease of white-tailed deer.

[3] Sayler KA, Dow C, Wisely SM (2016). Facts about wildlife diseases: hemorrhagic fever in white-tailed deer.

[4] Couvillion CE, Nettles VF, Davidson WR, Pearson JE, Gustafson GA. Hemorrhagic disease among white-tailed deer in the Southeast from 1971 through 1980. Proc US Anim Health Assoc. 1981;85:522–37.

[5] Savini G, Afonso A, Mellor P, Aradaib I, Yadin H, Sanaa M (2011). Epizootic heamorragic disease. Res Vet Sci.

[6] Nettles VF, Davidson WR, Stallknecht DE (1992). Surveillance for hemorrhagic disease in white-tailed deer and other wild ruminants, 1980-1989. Proc Annu Conf Southeast Assoc Fish Wildl Agencies.

[7] Brodie SJ, Bardsley KD, Diem K, Mecham JO, Norelius SE, Wilson WC (1998). Epizootic hemorrhagic disease: analysis of tissues by amplification and in situ hybridization reveals widespread Orbivirus infection at low copy numbers. J Virol.

[8] Ruder MG, Lysyk TJ, Stallknecht DE, Foil LD, Johnson DJ, Chase CC (2015). Transmission and epidemiology of bluetongue and epizootic hemorrhagic disease in North America: current perspectives, research gaps, and future directions. Vector-Borne Zoonotic Dis.

[9] Wisely SM, Sayler K (2016). Autogenous vaccine field trial for epizootic hemorrhagic disease virus and bluetongue virus does not result in high titer to homologous virus serotypes. Int Meet Emerg Dis Surveill IMED.

[10] Wisely S (2017). Vaccine field trial for EHDV does not produce antibody response [internet].

[11] Blackburn JK, McNyset KM, Curtis AJ, Hugh-Jones ME (2007). Modeling the geographic distribution of bacillus anthracis, the causative agent of anthrax disease, for the contiguous United States using predictive ecologic niche modeling. Am J Trop Med Hyg.

[12] Pfannenstiel RS, Mullens BA, Ruder MG, Zurek L, Cohnstaedt LW, Nayduch D (2015). Management of North American Culicoides biting midges: current knowledge and research needs. Vector-Borne Zoonotic Dis..

[13] Drolet BS, van Rijn P, Howerth EW, Beer M, Mertens PP (2015). A review of knowledge gaps and tools for Orbivirus research. Vector-Borne Zoonotic Dis..

[14] Cauvin A, Dinh ETN, Orange JP, Shuman RM, Blackburn JK, Wisely SM. Antibodies to Epizootic Hemorrhagic Disease Virus (EHDV) in Farmed and Wild Florida White-Tailed Deer (Odocoileus virginianus). J Wildl Dis. 2020;56(1):208–13. 31298969

[15] Cêtre-Sossah C, Roger M, Sailleau C, Rieau L, Zientara S, Bréard E (2014). Epizootic haemorrhagic disease virus in Reunion Island: evidence for the circulation of a new serotype and associated risk factors. Vet Microbiol.

[16] Reisen WK (2010). Landscape epidemiology of vector-borne diseases. Annu Rev Entomol.

[17] Morris LR, Proffitt KM, Asher V, Blackburn JK (2016). Elk resource selection and implications for anthrax management in Montana. J Wildl Manag.

[18] Brook RK, Wal EV, van Beest FM, McLachlan SM (2013). Evaluating use of cattle winter feeding areas by elk and white-tailed deer: implications for managing bovine tuberculosis transmission risk from the ground up. Prev Vet Med.

[19] Yaremych SA, Novak RJ, Raim AJ, Mankin PC, Warner RE. Home range and habitat use by American crows in relation to transmission of West Nile virus. Wilson J Ornithology. 2004;116(3):232–39. 10.1676/03-104.

[20] Janousek WM, Marra PP, Kilpatrick A (2014). Avian roosting behavior influences vector-host interactions for West Nile virus hosts. Parasit Vectors.

[21] McGregor BL, Stenn T, Sayler KA, Blosser EM, Blackburn JK, Wisely SM (2018). Host use patterns of Culicoides spp. biting midges at a big game preserve in Florida, U.S.a., and implications for the transmission of orbiviruses. Med Vet Entomol.

[22] Lyons A (2014). T-LoCoH for R: tutorial and users guide.

[23] Johnson DH (1980). The comparison of usage and availability measurements for evaluating resource preference. Ecology.

[24] Jennrich RI, Turner FB (1969). Measurement of non-circular home range. J Theor Biol.

[25] Laver PN, Kelly MJ (2008). A critical review of home range studies. J Wildl Manag.

[26] Stallknecht DE, Howerth EW (2004). Epidemiology of bluetongue and epizootic haemorrhagic disease in wildlife: surveillance methods. Vet Ital.

[27] Lyons A (2018). Package “tlocoh”.

[28] Lyons AJ, Turner WC, Getz WM (2013). Home range plus: a space-time characterization of movement over real landscapes. Mov Ecol.

[29] Schweiger AK, Schütz M, Anderwald P, Schaepman ME, Kneubühler M, Haller R (2015). Foraging ecology of three sympatric ungulate species – behavioural and resource maps indicate differences between chamois, ibex and red deer. Mov Ecol.

[30] ESRI (2014). ArcGIS 10.3.1 for Desktop.

[31] R Core Team. R: a language and environment for statistical computing. Vienna: R Foundation for Statistical Computing; 2014. https://scholar.google.com/citations?user=yvS1QUEAAAAJ&hl=en&oi=sra.

[32] Wilcoxon F (1945). Individual comparisons by ranking methods. Biom Bull.

[33] Mann HB, Whitney DR (1947). On a test of whether one of two random variables is stochastically larger than the other. Ann Math Stat.

[34] Wickham H (2016). ggplot2: elegant graphics for data analysis.

[35] Sorensen A, van Beest FM, Brook RK (2014). Impacts of wildlife baiting and supplemental feeding on infectious disease transmission risk: a synthesis of knowledge. Prev Vet Med..

[36] Cross PC, Heisey DM, Scurlock BM, Edwards WH, Ebinger MR, Brennan A (2010). Mapping brucellosis increases relative to elk density using hierarchical Bayesian models. PLoS One.

[37] Butler MJ, Teaschner AP, Ballard WB, McGee BK (2005). Wildlife ranching in North America—arguments, issues, and perspectives. Wildl Soc Bull.

[38] Miller R, Kaneene JB, Fitzgerald SD, Schmitt SM (2003). Evaluation of the influence of supplemental feeding of white-tailed deer (Odocoileus virginianus) on the prevalence of bovine tuberculosis in the Michigan wild deer population. J Wildl Dis.

[39] Maputla NW, Maruping NT, Chimimba CT, Ferreira SM (2015). Spatio-temporal separation between lions and leopards in the Kruger National Park and the Timbavati private nature reserve, South Africa. Glob Ecol Conserv.

