# Supplementary material for: Living la Vida T-LoCoH: site fidelity of Florida ranched and wild white-tailed deer (Odocoileus virginianus) during the epizootic hemorrhagic disease virus (EHDV) transmission period
Source: Mov Ecol. 2020 Mar 16;8:14. doi: 10.1186/s40462-020-00200-2 (PMC7076934; doi:10.1186/s40462-020-00200-2)
Supplement: Supplementary file 1 — Additional file 1: Table S1. Deer collared for this study. †30 Apr – 30 Oct = 183 days. ×Individuals excluded from home range behavior analysis. ^Individuals collared beyond 31 Oct 2017. [file 40462_2020_200_MOESM1_ESM.docx]

**Table S1. Deer collared for this study.** ^†^30 Apr – 30 Oct = 183 days. ^×^Individuals excluded from home range behavior analysis. ^Individuals collared beyond 31 Oct 2017.

| **Deer ID** | **Sex** | **Wild or Ranched** | **Start Date** | **End Date** | **Collar Type** | **# days recorded during EHD risk period^†^** |
| --- | --- | --- | --- | --- | --- | --- |
| OV063 | F | Ranched | 2016-04-13 | 2016-10-03 | ATS Neolink | 155 |
| OV061 | F | Ranched | 2016-04-13 | 2016-09-27 | ATS Neolink | 149 |
| OV069 | F | Ranched | 2016-04-14 | 2016-10-04 | ATS Neolink | 156 |
| OV070 | F | Ranched | 2016-04-14 | 2016-09-22 | Lotek 3300S | 144 |
| OV068*^×^* | F | Ranched | 2016-04-14 | 2016-07-20 | Lotek 3300S | 80 |
| OV064 | F | Ranched | 2016-04-14 | 2016-09-17 | ATS Neolink | 152 |
| OV059 | F | Ranched | 2016-04-13 | 2016-09-21 | ATS Neolink | 143 |
| OV102*^×^* | F | Wild | 2016-06-03 | 2016-06-29 | ATS Neolink | 26 |
| OV165 | F | Wild | 2016-05-24 | 2016-11-10 | ATS Neolink | 160 |
| OV154 | F | Wild | 2016-06-01 | 2017-12-02 | ATS Neolink | 152 |
| OV155*^×^* | F | Wild | 2016-06-01 | 2016-06-26 | ATS Neolink | 24 |
| OV168 | F | Wild | 2016-06-22 | 2017-08-22 | ATS Neolink | 131 |
| OV067 | M | Ranched | 2016-04-14 | 2016-09-02 | Lotek 3300L | 102 |
| OV074 | M | Ranched | 2016-04-15 | 2016-09-21 | Lotek 3300L | 143 |
| OV073 | M | Ranched | 2016-04-15 | 2016-09-23 | Lotek 3300L | 145 |
| OV066 | M | Ranched | 2016-04-14 | 2016-08-03 | Lotek 3300S | 94 |
| OV065 | M | Ranched | 2016-04-14 | 2016-09-21 | Lotek 3300S | 143 |
| OV062 | M | Ranched | 2016-04-13 | 2016-09-21 | Lotek 3300L | 143 |
| OV071 | M | Ranched | 2016-04-14 | 2016-09-22 | Lotek 3300L | 144 |
| OV072 | M | Ranched | 2016-04-14 | 2016-09-21 | Lotek 3300S | 143 |
| OV169 | M | Wild | 2016-06-23 | 2017-10-27 | ATS Neolink | 130 |
| OV166 | M | Wild | 2016-06-21 | 2016-11-08 | ATS Neolink | 132 |
| OV167 | M | Wild | 2016-06-20 | 2017-10-24 | ATS Neolink | 133 |
| OV490 | F | Ranched | 2017-04-13 | 2017-10-02 | ATS Neolink | 154 |
| OV491 | F | Ranched | 2017-04-13 | 2017-10-02 | Lotek 3300S | 154 |
| OV486 | F | Ranched | 2017-04-12 | 2017-10-02 | ATS Neolink | 154 |
| OV154 | F | Wild | 2016-06-01 | ^ | ATS Neolink | 152 |
| OV168*^×^* | F | Wild | 2016-06-22 | 2017-08-22 | ATS Neolink | 61 |
| OV522 | F | Wild | 2017-05-29 | ^ | ATS Neolink | 155 |
| OV450 | F | Wild | 2017-02-16 | ^ | ATS Neolink | 183 |
| OV427 | F | Wild | 2017-01-25 | 2017-10-30 | ATS Neolink | 183 |
| OV454 | F | Wild | 2017-02-24 | ^ | ATS Neolink | 183 |
| OV547*^×^* | F | Wild | 2017-02-27 | 2017-05-26 | ATS Neolink | 25 |
| OV428 | F | Wild | 2017-01-24 | ^ | ATS Neolink | 183 |
| OV446 | F | Wild | 2017-02-08 | ^ | ATS Neolink | 183 |
| OV505 | F | Wild | 2017-05-15 | ^ | ATS Neolink | 169 |
| OV430 | F | Wild | 2017-01-27 | ^ | ATS Neolink | 183 |
| OV093 | M | Ranched | 2017-04-12 | 2017-10-02 | ATS Neolink | 154 |
| OV065 | M | Ranched | 2017-04-14 | 2017-09-23 | ATS Neolink | 145 |
| OV489 | M | Ranched | 2017-04-12 | 2017-10-02 | ATS Neolink | 154 |
| OV169 | M | Wild | 2016-06-23 | 2017-10-27 | ATS Neolink | 179 |
| OV167 | M | Wild | 2016-06-20 | 2017-10-24 | ATS Neolink | 177 |
| OV464 | M | Wild | 2017-03-01 | ^ | ATS Neolink | 183 |
| OV435 | M | Wild | 2017-02-06 | ^ | ATS Neolink | 183 |
| OV460 | M | Wild | 2017-02-24 | ^ | ATS Neolink | 183 |
| OV555 | M | Wild | 2017-06-07 | ^ | ATS Neolink | 126 |
| OV431 | M | Wild | 2017-01-27 | ^ | ATS Neolink | 183 |
