# Supplementary material for: Living la Vida T-LoCoH: site fidelity of Florida ranched and wild white-tailed deer (Odocoileus virginianus) during the epizootic hemorrhagic disease virus (EHDV) transmission period
Source: Mov Ecol. 2020 Mar 16;8:14. doi: 10.1186/s40462-020-00200-2 (PMC7076934; doi:10.1186/s40462-020-00200-2)
Supplement: Supplementary file 2 — Additional file 2: Table S2. Sample sizes for testing significant differences in revisitation and duration between groups. [file 40462_2020_200_MOESM2_ESM.docx]

**Table S2. Sample sizes for testing significant differences in revisitation and duration between groups.**

| **Year** | **Group** | **Sample size** |
| --- | --- | --- |
| 2016 | Ranched females | 14,636 |
|  | Wild females | 7,626 |
|  | Ranched males | 17,838 |
|  | Wild males | 6,685 |
| 2017 | Ranched females | 8,131 |
|  | Wild females | 27,995 |
|  | Ranched males | 7,980 |
|  | Wild males | 21,523 |
